# Proposal Allocation Ratio as a Moderator of Interpersonal Responsibility Effects on Hostile Decision-Making in the Ultimatum Game

**DOI:** 10.3389/fpsyg.2017.01959

**Published:** 2017-11-14

**Authors:** Xinyu Gong, Ling-Xiang Xia, Yanlin Sun, Lei Guo, Vanessa C. Carpenter, Yuan Fang, Yunli Chen

**Affiliations:** ^1^Faculty of Psychology, Southwest University, Chongqing, China; ^2^Key Laboratory of Cognition and Personality, Ministry of Education, Southwest University, Chongqing, China; ^3^Key Laboratory of Sport Psychology, Tianjin University, Tianjin, China

**Keywords:** interpersonal responsibility, proposal allocation ratio, hostile decision-making, ultimatum game, Big Five personality

## Abstract

Interpersonal responsibility is an indigenous Chinese personality construct, which is regarded to have positive social functions. Two studies were designed to explore the relationship among interpersonal responsibility, proposal allocation ratio, and responders’ hostile decisions in an ultimatum game. Study 1 was a scenario study using a hypothetical ultimatum game with a valid sample of 551 high school students. Study 2 was an experimental study which recruited 54 undergraduate students to play the incentivized ultimatum game online. The results of the two studies showed a significantly negative correlation between interpersonal responsibility and responders’ rejection responses only when the proposal allocation ratio was 3:7. In addition, in Study 2, interpersonal responsibility had negative effects on responders’ rejection responses under the offer of 3:7, even after controlling for the Big Five personality traits. Taken together, proposal allocation ratio might moderate the effects of interpersonal responsibility on hostile decision-making in the ultimatum game. The social function of interpersonal responsibility might be beyond the Big Five.

## Introduction

Hostility refers to having negative attitudes and antagonistic actions toward others (Smith et al., 2004). Hostile behaviors are exhibited in many types of interpersonal interactions in daily life. Some social decisions could be regarded as hostile behaviors. For example, the rejection response of responders in an ultimatum game is considered hostile decision-making (Fetterman et al., 2014). The ultimatum game (Güth et al., 1982) is a widely used task to investigate decision-making in laboratory studies (Baumert et al., 2014; Thielmann and Hilbig, 2014). In the ultimatum game, two strangers share a sum of money. One party plays the role of the proposer, who makes an offer; the other party is the responder, who has the power to reject the offer. If the responder accepts the proposal, the money will be divided in accordance with the offer; when the proposal is rejected by the responder, both sides will get nothing. From the economic perspective, the responder’s rejection response (at the cost of sacrificing their own interests) is irrational, which reflects revenge and antagonism toward the proposer (Fetterman et al., 2014). Thus, the rejection response of responders in an ultimatum game could be used as an index of hostile behavior in social decision-making. To date, limited experimental studies (e.g., Fetterman et al., 2014) have explored hostility or aggression from the perspective of decision-making. The present study aimed to make contributions in the hostile decision-making domain.

Personality traits, especially the interpersonal traits, seem to influence both hostility and social decision-making. For instance, Honesty-Humility was found to be associated with both hostility (Ashton et al., 2014) and dictators’ allocations in the dictator game (Hilbig and Zettler, 2009). Several studies (Baumert et al., 2014; Hilbig et al., 2016) have explored the relationship between agreeableness and responders’ rejection decisions in the ultimatum game, but the results of these studies have been inconsistent. For example, Hilbig et al. (2013) and Thielmann et al. (2014) found that agreeableness could negatively predict the minimum amount which the responders would accept in the ultimatum game. However, Nguyen et al. (2011) asked participants to respond to 22 offers (2 offers of $5, 2 offers of $4, 6 offers of $3, 6 offers of $2, and 6 offers of $1), and each offer was a split of $10. He found that the relationship between Agreeableness and total rejection rate of the 22 offers was not significant. Thus, whether the interpersonal trait is associated with hostile decision-making warrants further exploration.

In addition, interpersonal traits described in the existing studies with respect to the relationship between personality and hostile decision-making were constructed by Western scholars and based on the Western culture. Western interpersonal traits may be complemented by the interpersonal traits derived from the non-Western culture (Cheung et al., 2011). Some interpersonal traits popular in the Chinese culture may have been ignored by Western personality theories (Gabrenya and Hwang, 1996). Thus, the current study sought to explore the relationship between interpersonal traits and hostile decision-making using a Chinese indigenous personality construct—Interpersonal responsibility.

Interpersonal responsibility is a recently proposed interpersonal trait that originated from the Chinese traditional culture and refers to being faithful and truthful to others (Xia, 2010; Xia et al., 2014a). It was found to be a protective personality factor of mental health and is beneficial for social communication (Xia et al., 2012a,b, 2014b). Hostility and aggression are always regarded as two of the psychological symptoms and negative social behaviors (Dodge, 1980; Ullrich et al., 2005). Presumably, interpersonal responsibility may resist hostile and aggressive behaviors. The results of the previous studies seem to support this notion. For instance, interpersonal responsibility was found to be negatively correlated with the hostility subscale of the Symptom Check List-90 (SCL-90) (Xia, 2010) and proactive and reactive aggression (Wu, 2016). Moreover, the relational schema is regarded as an important cognitive unit of interpersonal responsibility (Xia et al., 2012a). Individuals with high interpersonal responsibility usually have positive interpersonal attitudes toward others, whereas individuals with low interpersonal responsibility difficultly disengage from negative interpersonal information (Xia et al., 2014a). Hence, we assume that interpersonal responsibility may be negatively linked to hostile decision-making in the ultimatum game.

**FIGURE 1:**
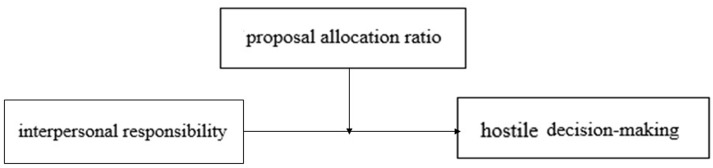
A model for the moderating effect of proposal allocation ratio between interpersonal responsibility and hostile decision-making.

As mentioned above, the results of the previous studies that explored the relationship between agreeableness and responders’ rejection decisions in the ultimatum game have been inconsistent. We speculate that the inconsistent results may be due to the different experimental conditions used in the previous studies. Specifically, the experimental condition of the required minimum amount may be more useful to reflect individual differences than the experiment condition of providing extensive categories of offers including some extreme offers (such as 10% of the total money offered). The previous studies (Sanfey, 2003; Koenigs and Tranel, 2007) have shown that responders would always accept the proposal when the offer is no less than 35% of the total money, while most of them are prone to reject the proposal when the offer is less than 20% of the total money. However, it is difficult to predict the responses when the offer is 20–35% of the total amount. Evidently, the reactions of most responders are quite consistent when the offer is more than 35% or less than 20%. In other words, responders’ behaviors would not differ under these offers. On the contrary, responders have different responses when the offer is 20–35% of the total amount. Specifically, the individual differences of responders’ reactions become greater under the offers of 20–35% of the total amount. Thus, it was hypothesized that proposal allocation ratio might moderate the relationship between personality (such as interpersonal responsibility) and responders’ hostile decision-making in the ultimatum game (**Figure 1**). Especially, when the proposal allocation ratio is 1:9, interpersonal responsibility may be not linked to hostile decision-making. In contrast, when the proposal is 3:7, interpersonal responsibility may have significant effects on responders’ rejections in the ultimatum game. In Study 1, the Interpersonal Self-Support Scale for Adolescent Students (ISSS-AS; Xia and Huang, 2008) and the hypothetical ultimatum game questionnaire were used to test this hypothesis.

Although both interpersonal responsibility and agreeableness are prosocial traits, interpersonal responsibility could not be included in agreeableness. The core characteristics (loyalty and faithfulness) of interpersonal responsibility are not held by agreeableness as honest-humility in the HEXACO personality model (a six-dimensional framework for personality structure) is different from agreeableness in the Big Five personality model. In addition, our prior empirical studies have shown that interpersonal responsibility has some positive functions beyond the Big Five. For example, interpersonal responsibility negatively predicted depression (Xia et al., 2012a) and interpersonal trust (Shen et al., 2016) even after controlling for the Big Five personality traits. Thus, it is very likely that interpersonal responsibility may play some positive roles in hostile decision-making, which may be independent of the present interpersonal traits constructed by western scholars. Therefore, the secondary purpose of this study is to explore whether interpersonal responsibility has specific effects on responders’ hostile decision-making beyond the effect of the Big Five personality traits when the proposal allocation ratio is 3:7 in the ultimatum game. In Study 2, the Interpersonal Self-Support Scale for Undergraduate Students (ISSS-US; Shen et al., 2016), the NEO Five-Factor Inventory (NEO-FFI), and the incentivized ultimatum game were used to further investigate the moderating effect of proposal allocation ratio on the relationship between interpersonal responsibility and hostile decision-making after controlling for the Big Five personality traits.

## Study 1

In Study1, we sought to explore the relationships between interpersonal responsibility and hostile decision-making under two kinds of proposal allocation ratios. According to the preceding theoretical analysis, it was hypothesized that the negative correlation between interpersonal responsibility and responders’ rejections in the hypothetical ultimatum game would be significant under the offer of 3:7 allocation but not significant under the offer of 1:9 allocation.

The hypothetical and incentivized ultimatum game are the two basic paradigms of ultimatum game. Some studies (Zhang and Ortmann, 2014; Thielmann et al., 2016) suggest that the results of hypothetical economic game are always consistent with the incentivized game. Additionally, the hypothetical ultimatum game have been used to explore the relationship between personality and social behaviors in previous research (e.g., Thielmann and Hilbig, 2014; Thielmann et al., 2014). Compare with incentivized game, hypothetical ultimatum game is more convenient and economic. Thus, we try to test our hypothesis using the hypothetical ultimatum game at first.

### Materials and Methods

#### Participants and Procedure

The participants included 587 high school students. Thirty-six participants were excluded from further analyses because they failed to complete all the instruments. The valid sample included 551 participants (266 females and 285 males). The mean age was 17.18 years (*SD* = 1.12, range = 14 to 20). After providing consent and demographic information, the ISSS-AS and the hypothetical ultimatum game questionnaire were administered. The order of the ISSS-AS and the game questionnaire was counterbalanced.

The Ethics Committee of the Southwest University of China approved our study. Participants over the age of 16 years provided written informed consent. Written informed consent was obtained from all the parents of participants who were under the age of 16 years.

#### Materials

##### Interpersonal Responsibility Subscale

The Interpersonal Responsibility Subscale used in Study 1 is one of the subscale of the ISSS-AS (Xia and Huang, 2008). It is a self-report scale that assesses interpersonal responsibility (e.g., “I cannot keep secrets of my friend(s) from others”) (*M* = 4.18, *SD* = 0.53) with four items rated on a 5-point scale from 1 (completely disagree) to 5 (completely agree). The Coefficient α of the interpersonal responsibility subscale was 0.71 in this study.

##### Hypothetical Ultimatum Game Questionnaire

Hypothetical hostile decision-making was assessed using the hypothetical ultimatum game questionnaire. Participants were told to imagine that they were playing an allocation game with some other students online in the psychology lab. They could neither see nor negotiate with each other during the game. The game included many trials. For each trial, one student was designated as the proposer who decided how to split 10 Chinese Yuan (∼1.5 US dollars). The proposer was changed for each trial. Participants were designated as responders who could choose to accept or reject the offer. If they accepted the offer, 10 Chinese Yuan would be divided in accordance with the proposal; if they rejected the offer, 10 Chinese Yuan would be forfeited. There were two kinds of proposal allocation ratio: (1) Responder gets 3 Chinese Yuan, proposer keeps 7 Chinese Yuan (3:7 allocation); (2) Responder gets 1 Chinese Yuan, proposer keeps 9 Chinese Yuan (1:9 allocation). In order to check whether participants understood the rules of the game, the questionnaire required participants to write the amount they achieved according to their choice.

#### Data Analysis

All statistical analyses were computed using SPSS 22. The intended level of significance was *a* = 0.05.

Hostile decision-making was scored such that rejection response was coded as 1 and acceptance response was coded as 0. In order to investigate the relationship between interpersonal responsibility (continuous variable) and hostile decision-making (binary variable) under two kinds of proposal allocation ratio, the point-biserial correlation was used. Further, the difference between the two correlation coefficients was tested by Steiger’s Z-test using Fisher’s r-to-z transformation (Fisher, 1921) in order to examine the moderating effect of proposal allocation ratio.

### Results and Discussion

The negative correlation coefficients between interpersonal responsibility and responders’ rejections under the offer of 3:7 allocation and 1:9 allocation were -0.28 (*p* < 0.01) and -0.07 (*p >* 0.05), respectively. Fisher’s test showed that the correlation coefficient between interpersonal responsibility and responders’ rejections was stronger under the offer of 3:7 allocation than was under the offer of 1:9 allocation (*Z* = -4.53, *p* < 0.01). These results support our hypothesis and suggest that the relationship between interpersonal responsibility and responders’ hostile decision-making might be affected by proposal allocation ratio.

This study was an exploratory research in which hypothetical ultimatum game was used to explore the moderating effect of proposal allocation ratio on the relationships between interpersonal responsibility and hostile decision-making. The moderating effect was achieved for the first time, which should be replicated in future research. In addition, prior studies (Chater et al., 2008; Lönnqvist et al., 2011) showed that people may be more generous or tolerant under hypothetical situations than incentivized situations. It is likely that individuals with high interpersonal responsibility may become more tolerant in a hypothetical situation and are more likely to accept unfair allocations. Hence, it is worth discussing responders’ hostile decision-making in the incentivized ultimatum game.

## Study 2

In order to replicate the results of Study 1 and overcome its potential limitations, the experimental method of incentivized ultimatum game was used in Study 2, and more types of the offer (1:9, 2:8, 3:7, 4:6, and 5:5 allocations) were included. In addition, the Big Five personality traits were included to investigate the effect of interpersonal responsibility on hostile decision-making after controlling for the Big Five personality traits under the offer of 3:7 allocation.

Most of the responders tend to accept the proposal when the offer is more than 35% of the total money and reject the proposal when the offer is less than 20% (Sanfey, 2003; Koenigs and Tranel, 2007). Thus, it could be inferred that almost everyone would accept the proposal under the offer of 5:5 allocation. Almost everyone would reject the proposal under the offer of 1:9 allocation. There was no relationship between interpersonal responsibility and responders’ rejections under the offer of 5:5 and 1:9 allocations. When the proposal allocation ratio was 4:6 or 2:8, individual variations in rejection responses seemed to be small, and the relationship between interpersonal responsibility and responders’ rejection responses may be weak. However, we did not have a clear prediction regarding whether the correlation under the offers of 2:8 and 4:6 allocations was significant. When the proposal allocation ratio was 3:7, it was hypothesized that interpersonal responsibility would have a significant effect on rejection responses in the incentivized ultimatum game of the present study, as in the hypothetical game of Study 1.

### Materials and Methods

#### Participants

A total of104 undergraduate students were assessed using the Interpersonal Responsibility Subscale of ISSS-US (Shen et al., 2016) at first.

Examining the differences in behaviors in experimental tasks between the high and low group on a personality variable to confirm the relationship between personality variable and behavior is a common method as is shown in literature (e.g., Mccleery and Goodwin, 2001; Li and Yang, 2013). The criterion to determine the cut-offs of the high and low group on a personality variable seem to be different and ambiguous in the prior studies (e.g., Van Hemert et al., 1993; Li and Yang, 2013; Chen et al., 2015). The present study refers to the upper and lower subgroups each containing 27% of the total group, which is quite a common cut-off in item analysis (Ayas and Sak, 2014; Ferrando, 2015) to determine the high and low group of interpersonal responsibility. After sorting participants’ scores on the Interpersonal Responsibility Subscale in descending order, 28 students (top 27%; *M_High_* = 4.43, *SD_High_* = 0.30) were selected and assigned to the high interpersonal responsibility group, and 28 students (bottom 27%; *M_Low_* = 3.48, *SD_Low_* = 0.32) in the low interpersonal responsibility group. Then, these 56 students were required to complete the experiment in exchange for a small monetary amount.

Prior to participation in the experimental tasks, the NEO-FFI was administrated to all 56 participants. However, data of two students from high interpersonal responsibility group were excluded because they failed to complete the NEO-FFI. Of the participants who reported their demographics, 24 were male and 30 were female (age 18–23 years, *M* = 19.56, *SD* = 1.28).

#### Measures

##### Interpersonal Responsibility Subscale

Interpersonal Responsibility Subscale used in Study 2 was a subscale of the ISSS-US (Shen et al., 2016), which was developed from ISSS-AS (Xia and Huang, 2008). It consists of eight items to assess interpersonal responsibility (e.g., “I never give others empty promises”; *M* = 3.94, *SD* = 0.57). Participants responded to each item on a scale ranging from 1 (completely disagree) to 5 (completely agree). The Coefficient α of the Interpersonal Responsibility Subscale was 0.75 in this study.

##### NEO Five-Factor Inventory

NEO Five-Factor Inventory is a simplified version of NEO Personality Inventory (NEO-PI; McCrae and Costa, 1992). It is a self-report scale with 60 items rated on a five-point Likert scale from 1 (completely disagree) to 5 (completely agree), which is comprised of five subscales: Neuroticism (*M* = 2.97, *SD* = 0.63), Extraversion (*M* = 3.43, *SD* = 0.50), Openness (*M* = 3.47, *SD* = 0.44), Agreeableness (*M* = 3.55, *SD* = 0.41), and Conscientiousness (*M* = 3.42, *SD* = 0.43). The Coefficient α of each subscale was 0.87, 0.81, 0.65, 0.65, and 0.75 respectively in this study.

#### Design and Procedure

The experiment had a 2 × 5 mixed design with covariates. Interpersonal responsibility (high and low) was a between-subjects variable, and proposal allocation ratio (5:5, 4:6, 3:7, 2:8, and 1:9) was a within-subjects variable, with Neuroticism, Extraversion, Openness, Agreeableness, and Conscientiousness as covariates. The dependent variable was responders’ hostile decision-making in the ultimatum game and its specific indicator was the rejection rate.

On arrival at the lab, participants were asked to sign an informed consent and fill a form about demographic information. Then, participants were assigned to complete the ultimatum game in the computer room. In order to increase the authenticity of the experiment, participants were told to play games online with students in the adjacent room. Moreover, all the students were to be paid according to their performances at the end of the game, by randomly selecting a trial response to garner their payment, accompanied by the base rate of 5 Chinese Yuan.

At the beginning of the experiment, participants were informed that the game comprised of two stages and each stage had 20 trials. In each trial, they shared 10 Chinese Yuan (∼1.5 US dollars) with one of the students in the adjacent room. One student played the role of the proposer who could present their proposal. The other student was designated as the responder who had the opportunity to reject or accept the offer. When they accepted the offer, 10 Chinese Yuan were divided in accordance with the proposal. When they rejected the offer, both sides got nothing. Participants were informed that in one stage, they would be proposers who would put forward their proposals to 20 different responders, and in the other stage, they would be responders who would receive 20 offers from 20 different proposers. The sequence of the two stages was randomly arranged by the computer.

**FIGURE 2:**
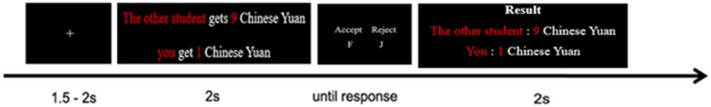
In this trial, the responder accepted the offer, thus the money was split between the two as proposed. Note that in the original task, all information presented was written in Chinese.

However, there was only one stage and everyone was selected to play the role of the responder. The computer program which was prepared in advance made 20 offers: 4 each of 5 types (5, 4, 3, 2, or 1 Chinese Yuan offered). In each trial, the participants first saw the interface of a central fixation cross for 1.5–2 s (randomized across trials). Next, the participants saw the proposal allocation ratio for 2 s (e.g., “The other student gets 9 Chinese Yuan, while you get 1 Chinese Yuan.”) on the screen. Then, the participants considered the offer with unlimited time [Accept (press the key “F”) or Reject (press the key “J”)] and pressed the key. Lastly, the participants saw the outcome based on his/her responses for 2 s (e.g., “You both get 0 Chinese Yuan” if the offer was rejected or “You get 1 Chinese Yuan, while the other student gets 9 Chinese Yuan” if the offer was accepted). The single experimental trial is presented in **Figure 2**.

There were three reasons for using such a procedure. First, the purpose of this study was simply to investigate responders’ hostile decision-making. Second, if participants were instructed to be responders all the time before the game, it could be procedural injustice (Dulebohn et al., 2009) and was very likely to induce dissatisfaction, resulting in more rejection responses. Third, it was easy for participants to suspect the authenticity of the experiment if they were told to merely play the role of the responder. Therefore, it was reasonable to set such procedures which could separate procedural injustice from distributive injustice to some extent.

After participants finished the game, they were debriefed. According to their self-report, all the participants believed that they were interacting with human proposers during the game. Then, they were told about the purpose of the experiment and the reason for setting such a game procedure. Finally, all the participants were remunerated with 10 Chinese Yuan regardless of their performance.

#### Data Analysis

All statistics were computed using SPSS 22. The intended level of significance was *a* = 0.05.

First, to test the role of interpersonal responsibility on hostile decision-making under different proposal allocation ratios, data were analyzed using 2 (interpersonal responsibility: high vs. low; between-participants) × 5 (proposal allocation ratio: 5:5 vs. 4:6 vs. 3:7 vs. 2:8 vs. 1:9; within-participants) mixed-factorial ANOVA. Then, we conducted a repeated measures ANCOVA with the Big Five personality traits as covariates to further investigate the moderating effect of proposal allocation ratios on the relationship between interpersonal responsibility and the hostile decision-making, after controlling for the Big Five personality traits. All ANCOVAs are presented with η^2^ as measure of effect sizes.

### Results and Discussion

The correlations of all variables are shown in **Table 1**.

**Table 1:** Inter-correlation for the variables included in Study 2.

	1	2	3	4	5	6	7	8	9	10	11
IR	-									
N	-0.28*	-									
E	0.06	-0.36**	-								
O	0.10	-0.10	0.17	-							
A	0.36**	-0.30*	0.19	0.12	-						
C	0.52**	-0.34*	0.38**	0.28*	0.31^∗^	-					
5:5	-0.02	-0.12	-0.25	-0.02	-0.35**	-0.03	-				
4:6	0.06	-0.12	-0.24	0.00	-0.28*	0.04	0.47**	-			
3:7	-0.43*	-0.01	-0.29*	-0.23	-0.12	-0.24	0.22	0.41**	-		
2:8	-0.20	0.13	0.02	-0.03	-0.00	-0.09	0.11	0.26	0.52**	-	
1:9	-0.20	0.07	-0.05	-0.10	0.01	-0.07	0.06	0.16	0.35*	0.58**	-

*IR, interpersonal responsibility; N, Neuroticism; E, Extraversion; O, Openness. A, Agreeableness; C, Conscientiousness; 5:5, the rejection rate under the offer of *5:5* allocation; 4:6, the rejection rate under the offer of 4:6 allocation; 3:7, the rejection rate under the offer of 3:7 allocation; 2:8, the rejection rate under the offer of 2:8 allocation; 1:9, the rejection rate under the offer of 1:9 allocation. *N* = 54; ^∗^*p* < 0.05; ^∗∗^*p* < 0.01*.

Mauchly’s test of sphericity for the ANOVA with interpersonal responsibility group and proposal allocation ratio as the independent variable was statistically significant; equal variances were not assumed. Therefore, Greenhouse and Geisser corrections for *F*-ratio were used. The results of the mixed-factorial ANOVA revealed that the main effect of proposal allocation ratio was significant, *F*_3.29/171.24_ = 117.57, *p* < 0.01, η^2^ = 0.69. The *post hoc* test with Bonferroni-adjusted multiple comparisons indicated that the rejection rate under the offer of 5:5 allocation (*M*_5:5_ = 0.02, *SD*_5:5_ = 0.14) differed significantly from the rejection rate under the offer of 3:7 allocation (*M*_3:7_ = 0.44, *SD*_3:7_ = 0.42), 2:8 allocation (*M*_2:8_ = 0.75, *SD*_2:8_ = 0.38), and 1:9 allocation (*M*_1:9_ = 0.90, *SD*_1:9_ = 0.27), all *p*s < 0.01. There was no significant difference between the rejection rate under the offer of 4:6 allocation (*M*_4:6_ = 0.13, *SD*_4:6_ = 0.31) and that under the offer of 5:5 allocation (*M*_5:5_ = 0.02, *SD*_5:5_ = 0.14), *p* = 0.068. The main effect of interpersonal responsibility was not significant, *F*_1/52_ = 3.70, *p* = 0.06. More importantly, we found that the predicted interaction between interpersonal responsibility and proposal allocation ratio was significant, *F*_3.29/171.24_ = 4.07, *p* < 0.01, η^2^ = 0.07.

**FIGURE 3:**
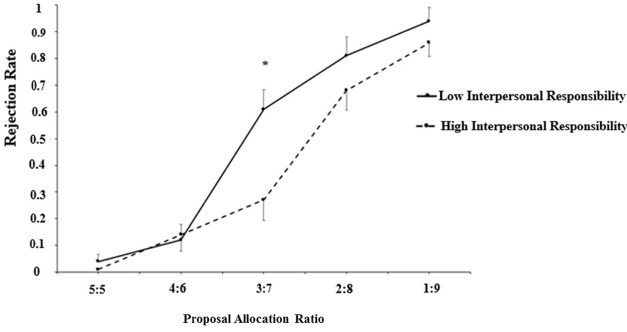
When the proposal allocation ratio was 3:7, interpersonal responsibility played a more important role on responders’ hostile decision-making, which was reflected by rejection rate, whereas when the proposal allocation ratio was 5:5, 4:6.2:8 or 1:9, interpersonal responsibility had little effect. The meaning of “^∗^” was that there was a significant difference between individuals with high and low interpersonal responsibility when the proposal allocation ratio was 3:7.

Simple effects analyses revealed (**Figure 3**), as expected, that when the proposal allocation ratio was 3:7, high interpersonal responsibility participants (*M*_3:7_ = 0.27, *SD*_3:7_ = 0.35) showed lower rejection rates than low interpersonal responsibility participants (*M*_3:7_ = 0.61, *SD*_3:7_ = 0.42), *F*_1/52_ = 10.11, *p* < 0.01, η^2^ = 0.16. When the proposal allocation ratio was 5:5 (*F*_1/52_ < 1), 4:6 (*F*_1/52_ < 1), 2:8 (*F*_1/52_ = 1.62, *p* = 0.21), or 1:9 (*F*_1/52_ = 1.26, *p* = 0.27), there was no difference between high (*M*_5:5_ = 0.01, *SD*_5:5_ = 0.05; *M*_4:6_ = 0.14, *SD*_4:6_ = 0.31; *M*_2:8_ = 0.68, *SD*_2:8_ = 0.44; *M*_1:9_ = 0.86, *SD*_1:9_ = 0.33) and low interpersonal responsibility participants (*M*_5:5_ = 0.04, *SD*_5:5_ = 0.19; *M*_4:6_ = 0.12, *SD*_4:6_ = 0.32; *M*_2:8_ = 0.81, *SD*_2:8_ = 0.30; *M*_1:9_ = 0.94, *SD*_1:9_ = 0.19).

Mauchly’s test of sphericity for the ANCOVA (with interpersonal responsibility group and proposal allocation ratio as independent variable, and the Big Five personality traits as covariates) was statistically significant; equal variances were not assumed. Therefore, Greenhouse and Geisser corrections for *F*-ratio were used. The results of ANCOVA showed that there was no significant interaction between proposal allocation ratio and Agreeableness (*F*_3.38/159.02_ = 1.73, *p* = 0.16), Neuroticism (*F*_3.38/159.02_ < 1), Openness *(F*_3.38/159.02_ < 1), Conscientiousness (*F*_3.38/159.02_ < 1), and Extraversion (*F*_3.38/159.02_ = 1.57, *p* = 0.19). Thus, assumptions of homogeneity of the Agreeableness, Neuroticism, Openness, Conscientiousness, and Extraversion regression slopes were tenable.

In addition, we found no main effect of interpersonal responsibility, *F*_1/47_ = 2.68, *p* = 0.11, proposal allocation ratio, *F*_3.38/159.02_ < 1, Agreeableness, *F*_1/47_ < 1, Openness, *F*_1/47_ < 1, Conscientiousness, *F*_1/47_ < 1, Neuroticism, *F*_1/47_ = 1.29, *p* = 0.26, or Extraversion, *F*_1/47_ = 2.52, *p* = 0.12. However, as predicted, we also found a significant interaction between proposal allocation ratio and interpersonal responsibility, *F*_3.38/159.02_ = 3.93, *p* < 0.01, η^2^ = 0.08. Simple effects analyses revealed that when the proposal allocation ratio was 3:7, high interpersonal responsibility participants showed lower rejection rates than low interpersonal responsibility participants, *F*_1/47_ = 8.53, *p* < 0.01, η^2^ = 0.15. When the proposal allocation ratio was 5:5 (*F*_1/47_ < 1, 4:6, *F*_1/47_ < 1), 2:8 (*F*_1/47_ = 1.51, *p* = 0.23), or 1:9 (*F*_1/47_ = 1.28, *p* = 0.26), there was no difference between high interpersonal responsibility group and low interpersonal responsibility group.

These results supported our hypothesis that the proposal allocation ratio, as one kind of situational factor, moderated the effect of interpersonal responsibility on the hostile decision-making in the ultimatum game, even after controlling for the Big Five personality traits. As expected, most responders accepted the offer when the proposal allocation ratio was 5:5. Most responders made rejection responses, even by sacrificing their own interests, when the proposal allocation ratio was 1:9. In short, under the proposal allocation ratios of 5:5 and 1:9, people’s response was similar and the influence of interpersonal responsibility on decision-making was small. On the contrary, there was a significant difference between individuals with high and low interpersonal responsibility when the proposal allocation ratio was 3:7, which was consistent with the result of Study 1 that the negative correlation between interpersonal responsibility and hostile decision-making was salient under the offer of 3:7 allocation. Additionally, this finding was also similar to the results of existing research (Hilbig et al., 2013; Thielmann and Hilbig, 2014) that agreeableness negatively predicts the minimum amount that responders would accept in the ultimatum game. These results supported our hypotheses that the proposal allocation ratio of 3:7 provided the scope for behavioral variability; thus interpersonal responsibility could play an important role in hostile decision-making. Individuals with high interpersonal responsibility tended to accept the offer, but those with low interpersonal responsibility were inclined to reject the offer with hostility when the proposal allocation ratio was 3:7.

There was no significant difference between high and low interpersonal responsibility individuals when the proposal allocation ratios were 4:6 and 2:8 in the present study. These findings were consistent with the results of previous studies. For example, many studies (e.g., Boarini et al., 2009; Boksem and De Cremer, 2010) indicated that responders always perceived fairness and accepted the offer when the offer was over 35% or less than 20% of the total money, and most responders had inequity aversion and were likely to make rejection responses, even by sacrificing their own interests.

In addition, as expected, the effect of interpersonal responsibility on responders’ hostile decision-making under the offer of 3:7 allocation existed after controlling for the Big Five personality traits in Study 2, which provided new evidence that interpersonal responsibility derived from Chinese traditional culture could play a unique role in people’s psychological activities and behaviors, beyond the Big Five personality traits. This result was similar to the previous study that interpersonal responsibility could negatively predict depression (Xia et al., 2012a) and interpersonal trust (Shen et al., 2016), even after controlling for the Big Five personality traits. These results supported the idea that Western interpersonal traits should be complemented by the interpersonal traits derived from the Chinese culture (Gabrenya and Hwang, 1996; Cheung et al., 2011).

Some limitations should be noted in Study 2. First, we use high and low group on interpersonal responsibility to test our hypothesis. However, the dichotomization of a continuous variable may result in some statistical problems (MacCallum et al., 2002). Therefore, the present results should be tested in the future without splitting the participants on interpersonal responsibility. Second, in the previous research (e.g., Stillmaker and Kasser, 2013; Chen et al., 2015), where the participants were divided into high and low groups on a personality variable before the experimental task, the sample sizes were always around 30 participants for each group. However, only 54 participants were involved in our experiment and the sample in the present study seemed to be insufficient to test the effect of personality on social behavior. It is wise to determine the sample size by using the software G^∗^Power (Panday and Rauniar, 2016; Stirrat et al., 2016) in the future study. Third, the Cronbach’s alphas of the subscales of Agreeableness and Openness in NEO-FFI (both = 0.65) were lower than the common standard, which may influence the statistical power of the results of Study 2. Further study should use large samples to get a good measurement of the Big Five personality traits and replicate the results of Study 2.

## General Discussion

First, this article investigated whether proposal allocation ratio played a moderating role in the relationship between the recently proposed interpersonal responsibility (Xia, 2010), which represented the tendency to be faithful and truthful to others, and hostile decision-making in the ultimatum game. Second, it explored whether the relationship between interpersonal responsibility and responders’ hostile decision-making in the ultimatum game was salient under the offer of 3:7 allocation in the ultimatum game, even after controlling for the Big Five personality traits. These two corresponding concerns were supported by findings from the two studies.

Both our studies with different methods and samples indicated that only when the proposal allocation ratio was 3:7, individual’s rejection of the offer may be due to the level of individual’s interpersonal responsibility. There are three reasons why responders with high interpersonal responsibility tended to accept the offer of 3:7 allocation and those with low interpersonal responsibility tended to reject it. First, high interpersonal responsibility individuals possess positive relational schemas (Xia et al., 2014b) and positive interpersonal cognitions (Xia et al., 2012a) such that they would pay attention to positive interpersonal information and use positive attribution. Hence, high interpersonal responsibility individuals may consider the offer of 3:7 allocation positively and regard others to be friendly, making less hostile attributions. Second, previous studies have found that individuals with high interpersonal responsibility tend to trust others and are inclined to cooperate rather than engage in hostile behaviors (Insko et al., 2005; Thielmann and Hilbig, 2014). Third, high interpersonal responsibility individuals may have greater perspective-taking and consider others’ feelings such that they tend to tolerate others’ offenses including exploitation in order to maintain interpersonal harmony. In contrast, low interpersonal responsibility individuals may have lower-perspective taking and may not forgive proposers under the offer of 3:7 allocation in the ultimatum game (Rizkalla et al., 2008).

The moderating effect of proposal allocation ratio seems to be explained by the strong situation hypothesis (Mischel, 1977). The strong situation hypothesis is always used to explain the effect of situational factors on the relationship between personality and behavior. This notion indicates that situational strength may moderate the relationship between personality and behavior. Specifically, strong situations are uniformly encoded and usually provide clear signals concerning how to act, which may lead people to construe the events and act in a similar way, weakening the relationship between the trait and the behavior. On the contrary, weak situations often provide vague signals and cannot generate uniform expectancies about the desired response pattern, which may permit people to act in any way they want; hence, individual behaviors are more likely to reflect their relevant personality traits. Thus, the influence of personality on behavior is much stronger in the weak situation, but relatively weak in the strong situation (Cooper and Withey, 2009). The strong situation hypothesis had been supported by some studies (Mcdonald and Donnellan, 2012; Grant and Rothbard, 2013; Lozano, 2016).

The proposal from the proposer could be regarded as an important situational cue to the responder, and the reactions of responders seem to be determined by the type of proposal. Previous studies (e.g., Boarini et al., 2009) and our present studies suggest that almost every person rejects the offer of 1:9 allocation and accepts the offer of 5:5 allocation; however, the responses of responders were very different under the 3:7 allocation offer. Presumably, the offers of 1:9 or 5:5 allocations may construct a typical strong situation in the ultimatum game, because people seem to have uniform notions with respect to 1:9 or 5:5 allocations, and accepting fair proposals or resisting extremely unfair offers seems to be a clear universal value or general rule in our current society (Boarini et al., 2009; Boksem and De Cremer, 2010). In other words, the offers of 1:9 or 5:5 allocations are uniformly encoded, and provide clear signals regarding rejecting or accepting the offers. Thus, the reactions of responders are almost consistent. In contrast, the offer of 3:7 allocation may produce the typical weak situation, because it provides ambiguous signals with respect to whether the proposer is “bad” or should be punished by rejecting his/her offer (Halko et al., 2009; Gu et al., 2016). Although whether the offer of 3:7 allocation representing a weak situation and the offers of 5:5 and 1:9 allocations representing strong situations warrant future testing, our studies suggested that the strong situation hypothesis might be a useful approach to understand the inconsistent results with regard to the relationship between personality and responders’ rejections in the ultimatum game. In other words, the strong situation hypothesis may extend our understanding of how personality and situational factors jointly influence hostile decision-making in the ultimatum game.

In addition, we found that interpersonal responsibility had an effect independent of the Big Five personality traits on responders’ rejection decisions in the ultimatum game under the offer of 3:7 allocation. These results provided new empirical evidence for the viewpoint that interpersonal responsibility derived in Chinese culture played served positive functions in mental health beyond the known personality traits derived from personality theories proposed by Western scholars (Xia et al., 2014b). Furthermore, the current study extended the application of the Interpersonal Self-Support theory mentioned above from the field of mental health area to hostility and decision-making domains. Future studies should test whether the effects of interpersonal self-support traits such as interpersonal responsibility on other social behaviors is independent of the current personality traits such as the Big Five.

Our studies have made some contributions in several aspects. First, we may have found a new personality trait, interpersonal responsibility, which was related to both hostility and economic decision-making. Second, a few previous studies (e.g., Fetterman et al., 2014) on hostility or aggression have focused on the decision-making behavior. Moreover, regarding decision-making, although cooperative behaviors were discussed considerably (Chater et al., 2008; Emonds et al., 2014), few studies have investigated hostile behaviors. Particularly, little research has combined decision-making and hostility. In our study, responders’ rejection response in the ultimatum game was used as an index to measure the hostile behavior, which may complement the experimental paradigm of the hostility.

However, our present studies were subject to some limitations. First, although consistent results were obtained in the two studies, the ages of samples in the two studies were quite different. Additionally, all the participants in the research were students. Decision-making may be different in some other social groups. Hence, the results should be replicated in future research using other similar and different samples. Specifically, future study should use the college, middle school, and community sample to replicate the results of the present study. A second limitation was that our study merely examined one kind of situational cue: proposal allocation ratio, which could affect the relationship between personality and responders’ hostile decision-making in the ultimatum game. However, a study has shown that social distance might decrease responders’ sensitivity to fairness in the ultimatum game (Kim et al., 2013). Thus, more situational factors need to be investigated in future studies. Third, though the results of the present studies could be explained by the strong situation hypothesis, we did not design our studies to test the strong situation hypothesis. Future studies should be designed to test the strong situation hypothesis and relationships among personality, situations, and hostile decision-making in ultimatum game. Fourth, the mechanism underlying the impact of interpersonal responsibility on responders’ rejections under the offer of 3:7 allocation has not been explored in this research. Fifth, our study was merely a preliminary study to investigate the role of interpersonal responsibility and proposal allocation ratio on hostile decision-making after controlling for the Big Five personality traits.

## Ethics Statement

This study was carried out in accordance with the recommendations of “the guidelines of the International Committee of Medical Journal Editors, the Ethics Committee of Southwest University of China” with written informed consent from all subjects. All subjects gave written informed consent in accordance with the Declaration of Helsinki. The protocol was approved by “the Ethics Committee of Southwest University of China.”

## Author Contributions

All authors contributed to the development and design of the studies. XG and L-XX drafted the manuscript. YS, LG, YF, YC, and VC were involved in critically revising it. All authors approved the final version of the manuscript for submission.

## Conflict of Interest Statement

The authors declare that the research was conducted in the absence of any commercial or financial relationships that could be construed as a potential conflict of interest.
